# Scintigraphic evaluation of oesophageal transit during radiotherapy to the mediastinum

**DOI:** 10.1186/1471-230X-8-51

**Published:** 2008-11-05

**Authors:** Giuseppe Sasso, Pierfrancesco Rambaldi, Francesco S Sasso, Vincenzo Cuccurullo, Paola Murino, Paolo Puntieri, Hugo R Marsiglia, Luigi Mansi

**Affiliations:** 1 Radiation Oncology Department, Second University of Naples, Naples, Italy; 2 Nuclear Medicine Department, Second University of Naples, Naples, Italy; 3 Faculty of Medicine, Health and Molecular Sciences, James Cook University, Townsville Campus, Australia; 4 Radiation Oncology Department, "Centre Medical de Forcilles" Hospital, Ferolles-Attilly, France; 5 Radiation Oncology Department, Institut Gustave Roussy, Villejuif, France; 6 Radiation Oncology Division, IMO International Oncology Group, Madrid, Spain; 7 Faculty of Medicine and Surgery, University of Florence, Florence, Italy

## Abstract

**Background:**

To quantitatively evaluate radiation-induced impaired oesophageal transit with oesophageal transit scintigraphy and to assess the relationships between acute oesophagitis symptoms and dysmotility.

**Methods:**

Between January 1996 and November 1998, 11 patients affected by non-small-cell carcinoma of the lung not directly involving the oesophagus, requiring adjuvant external beam radiotherapy (RT) to the mediastinum were enrolled. Oesophageal transit scans with liquid and semisolid bolus were performed at three pre-defined times: before (T0) and during radiation at 10 Gy (T1) and 30 Gy (T2). Two parameters were obtained for evaluation: 1) mean transit time (MTT); and 2) ratio between peak activity and residual activity at 40 seconds (ER-40s). Acute radiation toxicity was scored according to the joint EORTC-RTOG criteria. Mean values with standard deviation were calculated for all parameters. Analysis of variance (ANOVA) tests and paired t-Tests for all values were performed.

**Results:**

An increase in the ER-40s from T0 to T1 or T2 was seen in 9 of 11 patients (82%). The mean ER-40s value for all patients increased from 0.8306 (T0) to 0.8612 (T1) and 0.8658 (T2). These differences were statistically significant (p < 0.05) in two paired t-Tests at T0 versus T2 time: overall mean ER-40s and upright ER-40s (p = 0.041 and p = 0.032, respectively). Seven patients (63%) showed a slight increase in the mean MTT value during irradiation but no statistically significant differences in MTT parameters were found between T0, T1 and T2 (p > 0.05).

**Conclusion:**

Using oesophageal scintigraphy we were able to detect early alterations of oesophageal transit during the third week of thoracic RT.

## Background

External beam RT to the mediastinum is generally recommended in the treatment of a variety of thoracic tumours, in either a curative or a palliative setting. The oesophagus is often included in the radiation treatment volume. Acute oesophagitis is therefore one of the most frequent side effects and, particularly with the current trend for combined modality therapy, can be severe enough to interrupt the planned course of radiation therapy. Its pathophysiology, however, remains poorly understood.

The main clinical signs of acute radiation oesophagitis are dysphagia and odynophagia [1] that appear approximately during the third week of conventionally fractionated RT (2 Gy/day, 5 days/week), with a median total dose of 30 Gy [2]. The presence of these symptoms, which are clinical manifestation of dyskinesia and mucositis respectively [3], suggests that radiation-induced acute oesophagitis might be associated with altered organ motility. This hypothesis was supported by the efficacy of prophylactic pharmacological therapy consisting of Domperidone (a prokinetic agent) and Nimesulide (a non-steroidal anti-inflammatory) in delaying the onset and reducing the duration of oesophageal side effects in patients undergoing mediastinal irradiation [4].

A variety of studies have shown that irradiation of the oesophagus causes early mucosal changes and late organ dysmotility, fibrosis and stricture [5]. There is, however, uncertainty over whether RT causes acute effects on oesophageal motility or transit.

A number of well-conducted studies have been performed in order to assess (with manometry, endoscopy, scintigraphy or radiography) the presence of impaired oesophageal motility subsequent to irradiation. The results, however, are conflicting, most likely reflecting differences in study methodology and patient selection criteria.

Radionuclide oesophageal transit studies have been shown to be effective in the evaluation of oesophageal motility disorders [6] even when compared with oesophageal manometry, the current gold standard technique [7,8]. The high sensitivity of this method in detecting impaired oesophageal motility in patients with systemic sclerosis has been well demonstrated [9].

The aim of this study was to evaluate oesophageal transit time parameters by oesophageal scintigraphy to test the hypothesis that patients undergoing mediastinal RT may develop early oesophageal dysmotility and to assess the relationships between acute oesophagitis symptoms and dysmotility.

## Methods

### Study population and radiotherapy course

Between January 1996 and November 1998, a study was conducted on 11 patients (all males, mean age 59 years, range 48–75) affected by non-small-cell lung cancer not directly involving the oesophagus, treated with adjuvant external beam RT to the mediastinum. No patients with diffuse advanced malignant disease were enrolled in the study as they may have motility disorders of their gastrointestinal tract in the absence of demonstrable structural lesions [10,11].

All patients were treated with multiple field techniques with computed treatment planning and were selectively enrolled because they were considered at high risk of developing clinical radiation oesophagitis due to at least 12 cm cranial-caudal length of oesophagus being included in the planning target volume (PTV). For all patients the dose prescribed to the PTV (including mediastinal nodes and adjacent normal oesophagus) was 46 Gy in 23 fractions of 2 Gy each over 4 and half weeks. No patient received concomitant or neoadjuvant chemotherapy.

### Symptom Assessment

The incidence of oesophagitis during RT was evaluated twice weekly on the basis of clinical-anamnestic monitoring with reference to the joint European Organisation for Research and Treatment of Cancer and Radiation Therapy Oncology Group (EORTC-RTOG) scoring criteria [12] (Table 1). In all patients, before irradiation, barium swallow and a preliminary clinical-anamnestic examination excluded morphological abnormalities while scintigraphy failed to reveal alterations of oesophageal transit prior to radiation treatment. Endoscopic studies were not performed during the radiation treatment because of their poor compliance in patients suffering from acute radiation oesophagitis.

**Table 1:** Joint EORTC-RTOG acute toxicity scoring criteria

**Score**	**Symptoms**	**Suggested Therapy**
**0**	absence of alterations of the basal state	
**1**	slight dysphagia and/or odynophagia	topic anaesthetic and/or no-narcotic analgesics, light diet
**2**	moderate dysphagia and/or odynophagia	narcotics analgesics, diet based on purées or liquids
**3**	severe dysphagia and/or odynophagia with dehydration and weight loss >15%	nasogastric small probe for nutrition, liquid infusion or hyper-caloric diet
**4**	complete occlusion/obstruction, ulceration, perforation, (fistulae)	

European Organisation for Research and Treatment of Cancer and Radiation Therapy Oncology Group (EORTC-RTOG)

### Oesophageal Scintigraphy

The oesophageal scintigraphy studies were performed at three pre-defined times: before the beginning (T0) and at the end of the first and the third week of the RT course with a delivered radiation dose to the oesophagus of 10 Gy (T1) and 30 Gy (T2).

Oesophageal scintigraphy was performed according to the standards suggested by Klein HA in his comprehensive review [13]. The procedure was conducted using both liquid and semi-solid bolus. The liquid bolus, consisting of 5 ml water marked with ^99m^Tc-DTPA (technetium-99 m diethylene triamine penta-acetic acid), was administered both in the upright and supine position while the semi-solid radioactive meal, consisting of 10 ml of whipped egg albumen marked with ^99m^Tc-DTPA to achieve an activity concentration of 37 MBq/mL, was used for the third acquirement, only in upright position. Labelling efficiency of the egg albumen mixed with ^99m^Tc-DTPA was 90%, and the radiolabel remained "fixed" on the semisolid meal for an immediate usage.

All subjects were examined after 6 hours of fasting.

Imaging was performed using a large-field-of-view gamma camera fitted with a low-energy general-purpose parallel-hole (LEAP) collimator (Orbiter 75, Siemens, Erlangen). Computer analysis was performed both with a dedicated on-line workstation and with a post-processing PC workstation.

For each acquisition, the gamma camera was positioned ahead of the patient in the anterior projection. The field of view covered an area including the hypopharynx, oesophagus and stomach.

Oesophageal scintigraphy was constituted of three different phases. Radioactive reference points were positioned at the level of the jugular fossa and xiphoid, using radiolabelled markers, during the first phase (90 seconds). Then, in the second phase (30 seconds), the computer was set to acquire a dynamic study comprising 60 half-second frames followed, in the last phase, by 10-seconds frames for the succeeding 9 minutes. The overall duration of the whole examination (sum of the three phases) was 11 minutes.

The subject was refrained from swallowing until prompted with the radioactive meal placed in the mouth. When the computer was activated for study acquisition, the patient was asked to perform a single swallow and to abstain from swallowing during the following acquisition period. The study was repeated and if there was evidence or signs of possible additional swallows, a drink of not-radiomarked water was administered to the patient, at the end of the study, to remove any residual activity in the oesophageal lumen, confirmed by visual assessment on the gamma camera's video.

Regions of interest (ROI) were outlined for the mouth, whole oesophagus and stomach.

**Figure 1:**
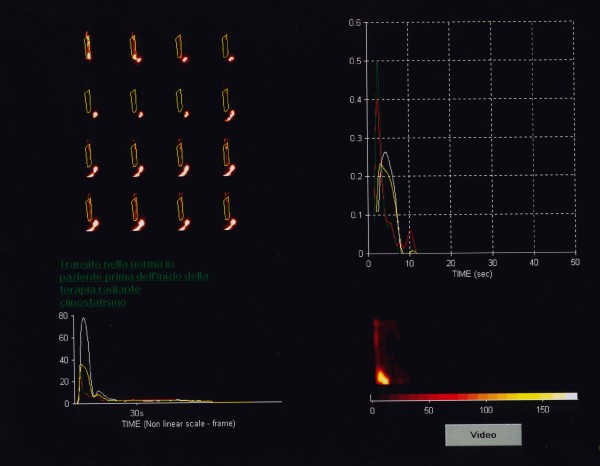
**Oesophageal scintigraphy: Dynamic condensed oesophageal images before radiation therapy (normal transit).**

**Figure 2:**
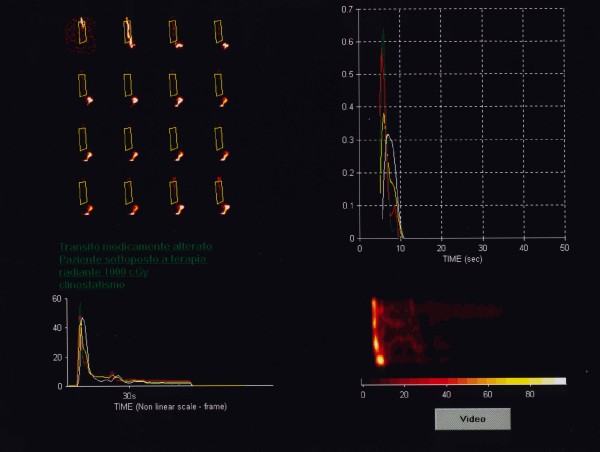
Oesophageal scintigraphy: Dynamic condensed oesophageal images during radiation therapy (slightly altered transit at 10 Gy).

**Figure 3:**
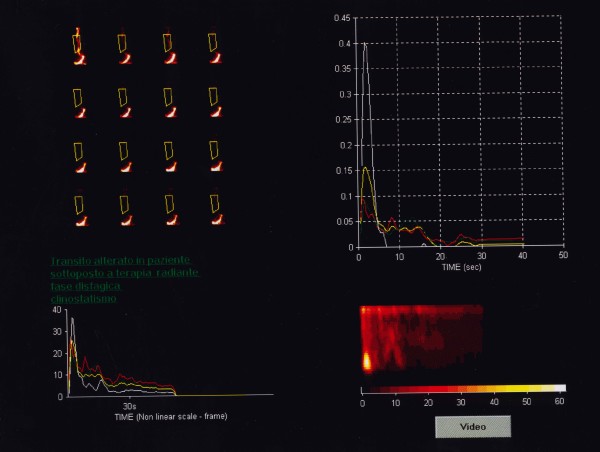
Oesophageal scintigraphy: Dynamic condensed oesophageal images during radiation therapy (distinctly altered transit at 30 Gy).

According to Klein and Wald [14] the study was processed using a standard, software program designed for the analysis of dynamic scintigraphy implemented with a proprietary algorithm for oesophageal transit evaluation with computer-generated condensed dynamic images reconstruction. As the transit process involves only the oesophageal cranio-caudal dimension (lateral motion is of no concern), the dynamic image data was condensed into a single image with one spatial dimension and one temporal dimension (vertical and horizontal respectively), allowing an easy calculation of the time-activity curves (Figure 1, 2 and 3).

After the calculation of the time-activity curves for each patient's ingestion, two parameters were obtained for evaluation: peak and 40 seconds residual activity ratio (ER-40s) and mean time of transit (MTT). These parameters were calculated for liquid bolus in upright and supine position and for semi-solid bolus in upright position only. The mean ER-40s and MTT values from the three different acquisitions for each patient and the mean ER-40s and MTT values from the whole database study set (mean of 33 acquisitions at 3 different times) were also calculated. The summary of the parameters obtained is described in Table 2.

**Table 2:** List of analysed parameters

1	ER-40s and MTT in upright position with liquid bolus for each patient.
2	ER-40s and MTT in supine position with liquid bolus for each patient.

3	ER-40s and MTT in upright position with semisolid bolus for each patient.

4	Mean ER-40s and MTT value from the 3 previously described parameters for each patient.

5	Mean of all the ER-40s and MTT values for the whole database study set (33 acquisition at 3 different times).

### Data Analysis

Mean values with standard deviation were calculated for all parameters. A paired t-test for each couple of data and for each type was performed. Also an analysis of variance (ANOVA) test was performed to assess the three sets of data (T0, T1 and T2) for each parameter. The study aimed to perform a repeated measurement of the sample population (to compare the measurements of the same people across time). Thus ANOVA for repeated measurements has been used in addition to "normal ANOVA".

## Results

The procedure was extremely well tolerated and all 11 patients were evaluable at T1 and T2. All patients complained of slight to moderate dysphagia and/or odynophagia at the end of the third week of the radiation course (T2, 30 Gy) but no patients developed grade 2 acute toxicity. Therefore no relationship was found between these symptoms and abnormal oesophageal transit in terms of time of appearance or degree of toxicity. There was no evidence of oesophageal gastric reflux in any examined patients.

Data from the study acquisition is shown in Table 3. Repeated measurements ANOVA test results (comparing T0, T1, and T2) are shown in Table 4.

**Table 3:** Study acquisition's data (values are in seconds for MTT and <ratio> for ER-40s)

Time		T0		T1		T2	
Patients	Study	ER-40s	MTT	ER-40s	MTT	ER-40s	MTT

1	upright	0.90	3.01	0.96	1.98	0.91	4.41
	supine	0.91	1.70	0.90	2.63	0.93	1.75
	upright ss	0.84	6.13	0.87	2.89	0.81	10.50
	mean	0.89	3.61	0.91	2.50	0.88	5.55
2	upright	0.80	2.00	0.88	2.39	0.89	2.03
	supine	0.82	4.68	0.91	9.98	0.88	2.03
	upright ss	0.90	8.12	0.91	1.80	0.92	2.11
	mean	0.84	4.93	0.90	4.73	0.89	2.05
3	upright	0.91	2.23	0.84	5.27	0.92	5.00
	supine	0.79	8.15	0.81	14.57	0.81	7.49
	upright ss	0.85	4.01	0.86	2.51	0.87	4.01
	mean	0.85	4.80	0.84	7.45	0.86	5.50
4	upright	0.86	2.03	0.90	3.00	0.92	4.00
	supine	0.84	3.06	0.93	4.34	0.94	6.00
	upright ss	0.95	1.87	0.93	2.87	0.90	4.00
	mean	0.88	2.32	0.92	3.41	0.92	4.67
5	upright	0.69	15.40	0.88	2.80	0.80	5.77
	supine	0.66	14.45	0.81	8.17	0.76	10.27
	upright ss	0.83	5.66	0.89	3.16	0.76	7.71
	mean	0.73	11.83	0.86	4.71	0.77	7.92
6	upright	0.70	13.30	0.78	5.40	0.72	12.59
	supine	0.82	3.47	0.68	12.51	0.60	14.68
	upright ss	0.63	13.90	0.66	13.70	0.75	8.34
	mean	0.72	10.22	0.71	10.54	0.69	11.87
7	upright	0.87	1.87	0.92	2.20	0.88	4.87
	supine	0.81	3.13	0.90	6.17	0.87	2.15
	upright ss	0.93	3.29	0.84	6.78	0.88	3.98
	mean	0.87	2.76	0.89	5.05	0.88	3.66
8	upright	0.97	2.02	0.90	2.70	0.93	1.13
	supine	0.65	15.39	0.83	2.23	0.92	8.95
	upright ss	0.93	1.95	0.93	1.92	0.91	2.11
	mean	0.85	6.45	0.89	2.28	0.92	4.07
9	upright	0.79	6.66	0.90	2.80	0.91	1.52
	supine	0.85	2.15	0.83	6.10	0.86	6.83
	upright ss	0.88	3.16	0.88	2.90	0.92	2.35
	mean	0.84	3.99	0.87	3.93	0.90	3.57
10	upright	0.84	3.26	0.91	2.86	0.91	4.40
	supine	0.52	16.44	0.67	21.29	0.87	6.80
	upright ss	0.88	4.82	0.89	2.82	0.88	4.00
	mean	0.75	8.17	0.82	8.99	0.89	5.07
11	upright	0.94	2.55	0.92	1.88	0.92	2.92
	supine	0.92	4.23	0.82	5.86	0.90	3.88
	upright ss	0.93	1.76	0.88	2.38	0.92	2.40
	mean	0.93	2.85	0.87	3.37	0.92	3.07

**Table 4:** Repeated measurements ANOVA (comparing T0, T1, and T2)

	p-value (based on Wilk's lambda)
Overall mean ER-40s	P = 0.117
Overall mean MTT	P = 0.815
Upright ER-40s	P = 0.113
Upright MTT	P = 0.244
Supine ER-40s	P = 0.383
Supine MTT	P = 0.556
Upright ss ER-40s	P = 0.986
Upright ss MTT	P = 0.501

Paired t-Tests results for T0 vs T1, T0 vs T2 and T1 vs T2 are shown in Table 5.

**Table 5:** Paired t-Tests results

	T0 vs T1	T0 vs T2	T1 vs T2
Overall mean ER-40s	P = 0.071	P = 0.041	P = 0.788
Overall mean MTT	P = 0.611	P = 0.522	P = 0.996
Upright ER-40s	P = 0.069	P = 0.032	P = 0.614
Upright MTT	P = 0.195	P = 0.658	P = 0.090
Supine ER-40s	P = 0.180	P = 0.159	P = 0.359
Supine MTT	P = 0.431	P = 0.752	P = 0.270
Upright ss ER-40s	P = 0.943	P = 0.868	P = 0.921
Upright ss MTT	P = 0.227	P = 0.765	P = 0.505

### Residual activity ratio at 40 seconds (ER-40s)

**Figure 4:**
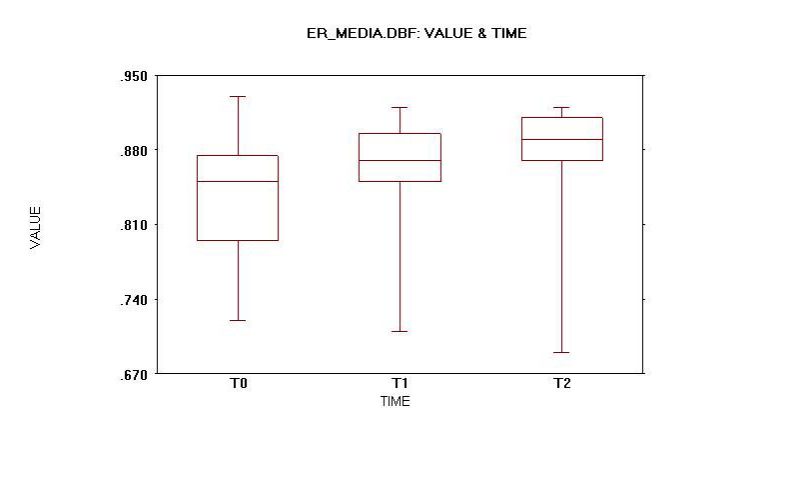
Oesophageal scintigraphy: Residual activity ratio at 40 seconds (ER-40s) mean values.

Data from supine and upright position study with liquid and semi-solid meal were available for all patients and for each of them a mean value of three data sets was also calculated (mean ER-40s, Fig. 4).

An increased mean ER-40s from T0 to at least one of the further studies was seen in 9 of 11 patients (81.8%). An increased ER-40s from T0 to at least one of the further studies was also seen in 9 of 11 patients (81.8%) for the acquisition with liquid bolus in upright and supine position while it was noted in only 7 of 11 patients (63.6%) with semi-solid meal.

The mean ER-40s for the whole database study set for all patients passed from a value of 0.8306 (SD 0.1052) at T0 to 0.8612 (SD 0.0739) at T1 and further increased to 0.8658 (SD 0.0759) at T2. The mean ER-40s for all patients passed from a value of 0.8318 (SD 0.0687) at T0 to 0.8618 (SD 0.0584) at T1 and further increased to 0.8655 (SD 0.0719) at T2. The mean upright position ER-40s for all patients passed from a value of 0.8427 (SD 0.0910) at T0 to 0.8900 (SD 0.0471) at T1 and to 0.8827 (SD 0.0648) at T2. The mean supine ER-40s for all patients passed from a value of 0.7809 (SD 0.1217) at T0 to 0.8264 (SD 0.0866) at T1 and further increased to 0.8491 (SD 0.0979) at T2. By contrast, the mean semi-solid bolus ER-40s for all patients passed from a value of 0.8682 (SD 0.0887) at T0 to 0.8673 (SD 0.0740) at T1 and further decreased to 0.8655 (SD 0.0633) at T2.

Thus at the 0.05 significance level all the differences previously described between the various mean ER-40s in supine and upright position with liquid and semi-solid bolus at T0, T1 and T2 were not significantly different (p > 0.05) at both the standard and repeated measurement ANOVA tests.

At paired t-Test analysis two tests were statistically significant at T0 vs T2 time comparison: respectively, Overall mean ER-40s (p = 0.041) and Upright ER-40s (p = 0.032).

### Mean Transit Time (MTT)

**Figure 5:**
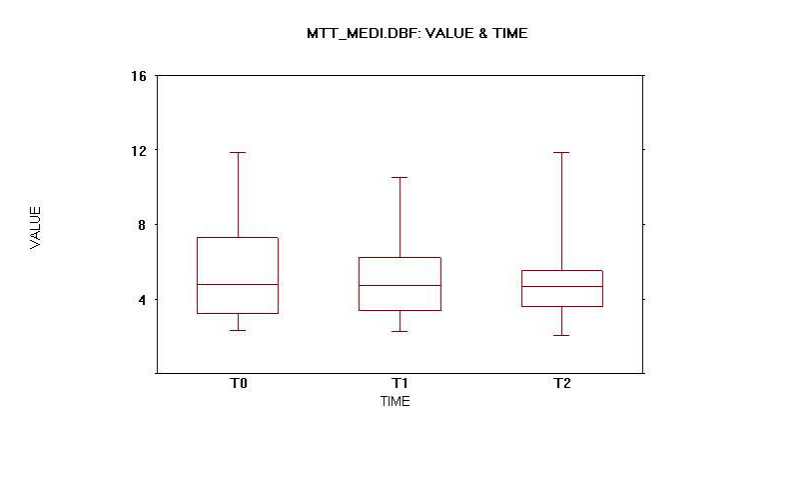
Oesophageal scintigraphy: Mean transit time (MTT) mean values.

Data from supine and upright position study with liquid and semi-solid meal were available for all patients and for each of them a mean value of three data sets was also calculated (mean MTT, Fig. 5).

An increased mean MTT from T0 to at least one of the further studies was seen in 7 of 11 patients (63.6%). An increased MTT from T0 to at least one of the further studies was also seen in 7 of 11 patients (63.6%) for the acquisition with liquid bolus in upright position and 8 of 11 (72.7%) patients for the supine position, while it was noted in only 6 of 11 patients (54.5%) with semi-solid meal.

The mean MTT for the whole database study set for all patients passed from a value of 5.6318 (SD 4.7392) at T0 to 5.1776 (SD 4.5231) at T1 and to 5.1812 (SD 3.3632) at T2. The mean MTT for all patients decreased from a value of 5.6300 (SD 3.1859) at T0 to 5.1782 (SD 2.6908) at T1 and to 5.1818 (SD 2.7048) at T2. The mean upright position MTT for all patients passed from a value of 4.9391 (SD 4.8675) at T0 to 3.0255 (SD 1.2001) at T1 and to 4.4218 (SD 3.1062) at T2. The mean supine MTT for all patients passed from a value of 6.9864 (SD 5.6917) at T0 to 8.5318 (SD 5.7274) at T1 and decreased to 6.4391 (SD 3.9604) at T2. The mean semi-solid bolus MTT for all patients passed from a value of 4.9700 (SD 3.5716) at T0 to 3.9755 (SD 3.4877) at T1 and then increased to 4.6827 (SD 2.8662) at T2.

At the 0.05, significance level, all differences previously described between the various mean MTT in supine and upright position with liquid and semi-solid bolus at T0, T1 and T2 were not significantly different (p > 0.05) for both ANOVA and paired t-Tests.

## Discussion

The relationships between acute and chronic radiation side effects are controversial and the prediction of their severity is difficult [15]. Although oesophageal symptoms are common during adjuvant thoracic RT, the effects of irradiation on oesophageal function are still to be assessed.

Oesophagitis arises in at least two-thirds of patients undergoing mediastinal RT. EORTC-RTOG grade 2 and grade 3 oesophagitis occurs in 12% and 3% of patients respectively [16]. In the majority of patients, it's onset is during the third week of the RT course. Any concomitant chemotherapy and simultaneous exposure of the organ to exogenous infectious agents by micro-traumatising agents (mainly some foods) increases the risk of radiation toxicity.

Around one third of patients develop clinical symptoms without histological changes [17] and endoscopic and radiographic identification and quantification of subtle disorders remains a problem.

Patients with clinical signs of acute radiation oesophagitis often have negative single-contrast oesophagograms [18] and only in a few positive cases was a thin indentation of the barium column demonstrated. Double contrast radiological examination may reveal a variable segment of oesophageal narrowing with multiple discrete ulcers or a distinctive granular appearance of the mucosa within a known irradiated volume, suggesting the diagnosis of radiation oesophagitis [19], but a quantitative evaluation of altered oesophageal transit cannot be assessed.

Endoscopic studies may show oedema and vulnerability of the mucosa without erosion in patients with clinical signs of radiation-induced oesophagitis [20], but additional dynamic visual analysis might be helpful in diagnosing early changes.

Oesophageal scintigraphy is a well-established nuclear imaging method for the detection of motility disorders that has been used in a variety of clinical situations such as systemic sclerosis, oesophageal spasm, achalasia [21], diabetes mellitus [22], reflux disease and dysphagia of unclear origin. It is a non-invasive diagnostic method with a low radiation dose and offers the possibility of quantitative analysis.

The clinical, value of radionuclide oesophageal transit measurements in relation to established oesophageal motility investigations, was assessed by de Caestecker et al. [23]. In their experience of 150 patients, the overall sensitivity in detecting oesophageal dysmotility was 75% for radionuclide transit measurements, 83% for manometry and 30% for conventional barium radiology. In 18 patients oesophageal scans identified abnormalities not detected by manometry. The authors concluded that radionuclide transit measurements were a useful test for patients with suspected oesophageal motility disorders, providing additional information which complemented oesophageal manometry, although it did have limitations as a screening test. The validity of non-invasive oesophageal transit scintigraphy to quantitatively assess gastrointestinal motor dysfunction has been supported by a number of other studies and reviews [24-27].

According to Holloway et al. [28], the main limitation of the radionuclide transit test may be a consequence of using a liquid bolus. This successfully identifies motor disorders characterized by defective peristaltic progression but fails to discover disorders in which peristalsis is intact, probably because of the small number of swallow sequences tested.

Dysphagia and odynophagia are the symptoms most commonly experienced by patients with acute radiation oesophagitis [1], but objective documentation of early dysphagia remains difficult. The rate of mild (grade 1) toxicity ranges between 60% and 100% in published series. Dysphagia and odynophagia are clinical manifestations of dyskinesia and mucositis, respectively, suggesting a potential role for oesophageal transit studies in providing prognostic data for early treatment phase evaluation of radiation oesophagitis.

In order to further clarify the pathogenesis of these sequelae, Yeoh et al. [29] used barium swallow, endoscopy, combined radionuclide scintigraphy and oesophageal manometry to evaluate eight patients before, during and after mediastinal irradiation for potentially curable intra-thoracic malignant disease. The authors concluded that post-radiation oesophageal symptoms are not a result of altered oesophageal motility or transit but may reflect increased mucosal sensitivity.

The absence of observed abnormal peristaltic response conflicts with the previous prospective study of LaManna et al. [30] suggesting that post-radiation oesophageal symptoms may reflect disordered oesophageal motility as evaluated by radionuclide oesophageal transit scintigraphy.

This discrepancy may be attributed to differences in methodology of the studies and patient selection criteria, both inducing prolongation of the oesophageal transit time in the LaManna et al. study. In the Yeoh et al. study, the absence of significant changes in oesophageal motility despite the development of symptoms may have been influenced by the inclusion of 3 patients (37%) with carcinoma of the breast. The dose to the oesophagus in those patients was approximately one third of the dose received by the other participants on the study. In addition, a number of patients had underlying abnormalities in oesophageal function which may have masked any small increase in oesophageal transit times.

Oesophageal scintigraphy has also been found to be a sensitive tool for the evaluation of dysphageal symptoms and quantification of the effect of local analgesic treatment during mediastinal RT in a study by Brandt-Mainz et al. [31].

The exact role of oesophageal transit scintigraphy in the evaluation of gastrointestinal motility disease remains controversial. It appears useful when oesophageal manometry is poorly tolerated, such as in patients suffering from acute radiation oesophagitis.

There is a large dependence of measurements on multiple variables such as the patient's position, projection, bolus consistency and volume, isotope, number of independent wet swallows, analytical parameters used, and consideration of age. [32]. Therefore a protocol based on both published data and accumulated local experience is recommended and normal ranges should be defined in one's own laboratory.

The emptying rate (ER) and the mean transit time (MTT) are among the most commonly used parameters for evaluation of oesophageal transit and have already been suggested for the assessment of radiation related oesophageal disorders (Brandt-Mainz et al.).

The originality of our study lies in the evaluation of oesophageal transit time parameters by a non-invasive and well-tolerated method of oesophageal scintigraphy. We hypothesised that patients submitted to mediastinal RT, whether symptomatic or not, might have early impaired oesophageal transit evaluable with this technique.

One of the main strengths of our study, when compared to previous studies, is the homogeneous sample population and treatment technique. All patients received 46 Gy to at least a 12 cm length of oesophagus with a uniform radiation technique and fields. Given the significant volume of oesophagus contained within the full dose region, all patients were at high risk of developing radiation-induced oesophagitis. All patients did, infact, develop grade 1 toxicity during the third week of treatment and all were symptomatic at the time of the T2 study. No patients suffered from grade 2 or 3 radiation oesophagitis. This was most likely due to the absence of concomitant chemotherapy and the lower total dose of irradiation used in the adjuvant setting.

An additional strength of our study is in the timing of oesophageal scintigraphy during the RT course. To our knowledge, this is the largest study of oesophageal transit during RT and the only one in which the scintigraphy is performed at the end of the first and the third week of RT, with the aim of assessing early alterations. The T2 study aimed to detect changes in oesophageal motility during or soon after the symptoms of oesophagitis would generally appear (i.e. around the third week). By contrast, the T1 study was performed after only one week of RT in an attempt to identify any earlier signs of dysmotility that may predict for the subsequent development of symptomatic acute oesophagitis. The reason to perform this early (T1) documentation of transit was also supported by studies of fractionated RT in dogs showing that gastrointestinal motility changes occur within 48 hours of the initiation of therapy [33,34].

A limitation of this study is the lack of a comparison with manometry, which has been considered the most sensitive method to detect impaired oesophageal motility and lower oesophageal sphincter dysfunction. Kjellen et al., however, have reported on the utility of oesophageal scintigraphy in 16 patients with symptomatic dysphagia but having normal manometry, acid perfusion, acid clearance and pH reflux tests [35]. They concluded that scintigraphy could be regarded as a valuable complement in the objective documentation of dysphagia when other diagnostic methods fail.

Our study found a trend from T0 to further studies in the mean ER-40s value and this was confirmed in the detailed analysis in 9 of 11 patients (81.8%). These differences were not statistically significant (p > 0.05) at ANOVA but two parameters, overall mean ER-40s (p = 0.041) and upright ER-40s (p = 0.032), had changes between the baseline study (T0) and the study performed after three weeks of treatment (T2) which were statistically significant at the paired t-Test analysis.

We acknowledge that the statistical significance of our results is limited despite our homogeneous sample population and treatment technique. However, in view of the high variation in measurements between people, a large sample size would be needed to draw further conclusions. We believe there would be little chance of detecting something more (ie. ANOVA or p < 0.002 with Bonferroni paired t-Tests) with further studies.

Finally, our study was designed about 10 years ago. Since then indications for adjuvant or radical treatment of non-small-cell lung cancer have changed resulting in a dramatic increase in the use of sequential and concomitant chemotherapy with conformal RT and a more limited volume of oesophagus being irradiated (though often to a higher dose). Therefore, it is unlikely that a similar subgroup of patients will be available in the near future for enrolment in a comparable clinical trial as further studies will most likely focus on the effects and changes due to intensive combined modality treatments.

## Conclusion

In conclusion, this is one of the few prospective studies examining the pathogenesis of oesophageal sequelae from mediastinal irradiation. Our study may be limited by the lack of endoscopic and manometric data, however, to our knowledge it is the largest and most homogeneous series of patients studied in this manner. Moreover, it is unique in studying patients prior to radiation exposure and early in the course of treatment before symptoms appear. Our results confirm that oesophageal scintigraphy can show early alterations of oesophageal transit during the third week of thoracic RT. We are aware that the statistically significance of our results is limited in spite of the best efforts to ensure a very homogeneous sample population and treatment technique. However, in view of the sample size needed for further conclusions and the very high variation of the measurements between people there is little chance to detect something more (ie. ANOVA or p < 0.002 with Bonferroni paired t-Tests) with further studies.

Finally, our study was designed about 10 years ago. Since then indications for adjuvant or radical treatment of non-small-cell lung cancer have changed resulting in a dramatic increase in the use of sequential and concomitant chemotherapy with radiation therapy and a more limited irradiation of the oesophagus, although with sometimes higher doses to small volume of the organ. Therefore it is unlikely that a similar subgroup of patients will be available in the near future for enrolment in a comparable clinical trial as further studies will most likely focus on the effects and changes due to intensive combined modality treatments.

## Competing interests

The authors declare that they have no competing interests.

## Authors' contributions

GS and PR conceived the study, participated in its design, carrying out and coordination. VC PM and PP carried out the study and drafted the manuscript. HRM drafted the manuscript and performed the statistical analysis. FSS and LM conceived the study, and participated in its design and coordination.

All authors read and approved the final manuscript.

## Pre-publication history

The pre-publication history for this paper can be accessed here:


